# Herd specific risk factors for *Mycoplasma hyopneumoniae* infections in suckling pigs at the age of weaning

**DOI:** 10.1186/1751-0147-55-30

**Published:** 2013-04-12

**Authors:** Heiko Nathues, Henrike Woeste, Stefanie Doehring, Anna S Fahrion, Marcus G Doherr, Elisabeth grosse Beilage

**Affiliations:** 1 Field Station for Epidemiology, University of Veterinary Medicine Hannover, Foundation, Buescheler Street 9, Bakum D-49456, Germany; 2 Veterinary Epidemiology, Economics and Public Health Group, Department of Production and Population Health, Royal Veterinary College, Hawkshead Lane, North Mymms, Hatfield, Hertfordshire, AL9 7TA, UK; 3 Veterinary Public Health Institute, Department of Clinical Research - Veterinary Public Health, Vetsuisse Faculty, University of Bern, Schwarzenburgstrasse 155, Liebefeld (BE), CH-3097, Switzerland

**Keywords:** *Mycoplasma hyopneumoniae*, Enzootic pneumonia, Suckling pig, Epidemiology, Risk factor analysis, Prevalence

## Abstract

**Background:**

*Mycoplasma hyopneumoniae* is the etiologic agent of enzootic pneumonia mainly occurring in fattening pigs. It is assumed that horizontal transmission of the pathogen during nursery and growing phase starts with few suckling pigs vertically infected by the sow. The aim of the present study was the exploration of the herd prevalence of *M. hyopneumoniae* infections in suckling pigs followed by an investigation of various herd specific factors for their potential of influencing the occurrence of this pathogen at the age of weaning.

**Results:**

In this cross-sectional study, 125 breeding herds were examined by taking nasal swabs from 20 suckling pigs in each herd. In total, 3.9% (98/2500) of all nasal swabs were tested positive for *M. hyopneumoniae* by real-time PCR. Piglets tested positive originated from 46 different herds resulting in an overall herd prevalence of 36.8% (46/125) for *M. hyopneumoniae* infection in pigs at the age of weaning. While the herds were epidemiologically characterized, the risk for demonstration of *M. hyopneumoniae* was significantly increased, when the number of purchased gilts per year was more than 120 (OR: 5.8), and when the number of farrowing pens per compartment was higher than 16 (OR: 3.3). In herds with a planned and segregated production, where groups of sows entered previously emptied farrowing units, the risk for demonstration of *M. hyopneumoniae* in piglets was higher in herds with two or four weeks between batches than in herds with one or three weeks between batches (OR: 2.7).

**Conclusions:**

In this cross-sectional study, several risk factors could be identified enhancing the probability of breeding herds to raise suckling pigs already infected with *M. hyopneumoniae* at the time of weaning. Interestingly, some factors (farrowing rhythm, gilt acclimatisation issues) were overlapping with those also influencing the seroprevalences among sows or the transmission of the pathogen between older age groups. Taking the multifactorial character of enzootic pneumonia into account, the results of this study substantiate that a comprehensive herd specific prevention programme is a prerequisite to reduce transmission of and disease caused by *M. hyopneumoniae*.

## Background

Despite more than four decades of attempts to control porcine enzootic pneumonia [1,2], which also included more than fifteen years of vaccination against *Mycoplasma hyopneumoniae*, the etiologic agent is still widespread in nearly all pig populations. Noteworthy, enzootic pneumonia has remained one of the most important respiratory diseases in growing and fattening pigs leading to significant economic impacts on pig production throughout the world.

In general, the pathogen is transmitted nose to nose by vertical route from sows to their offspring [3] or by very effective horizontal route between pen mates or pigs in the same compartment [4]. If the system of all-in/all-out is not consequently implemented in the different production stages, there are also frequent transmissions of *M. hyopneumoniae* from older, infected to younger, naïve pigs [5]. It is assumed that suckling pigs from gilts and young sows, which are shedding *M. hyopneumoniae* more often than middle aged sows, have a higher risk of becoming positive by vertical transmission [6]. Despite this, the seroprevalence of *M. hyopneumoniae* differ between endemically infected herds, which probably reflect the pathogen load of the herds. In some herds older sows are more often seropositive than younger ones [7]. Noteworthy, seropositivity is not necessarily correlated with the shedding of *M. hyopneumoniae*[6] and more research is required to determine how sow parity affects the shedding of *M. hyopneumoniae*[8].

Next to these risk factors considering the age of the sows, vaccination of sows against *M. hyopneumoniae* has been described potentially influencing the vertical transmission of the pathogen and course of enzootic pneumonia in growing pigs [9].

Above all, no other potential risk factors for the occurrence of *M. hyopneumoniae* in piglets at the age of weaning considering the management, hygiene measures, vaccination programmes and husbandry systems on herd level have been characterised in detail. Since it is assumed that horizontal transmission of *M. hyopneumoniae* during nursery and growing phase starts with few vertically infected piglets [10] knowledge about these risk factors seems to be a prerequisite for efficient control and prevention programmes.

The aim of the present study was the exploration of the herd prevalence of *M. hyopneumoniae* infections in suckling pigs followed by an investigation of various herd specific factors for their potential of influencing the occurrence of this pathogen at the age of weaning. For this purpose, a cross-sectional study was conducted, where herds were epidemiologically characterised and *M. hyopneumoniae* status of suckling pigs was assessed by examining nasal swabs with a highly sensitive real-time PCR. It was hypothesised that more information about factors triggering the vertical transmission of *M. hyopneumoniae* from sows to their offspring will also improve the knowledge about horizontal transmission.

## Methods

Data for this cross-sectional field study were collected between June 2009 and July 2010, thus covering all seasons of the year. All farm owners and veterinarians agreed on using the data from this study for publication except of their names and addresses. The collection of nasal swabs from pigs was not considered being an 'animal experiment according to the German Animal Welfare Act'. Nonetheless, the study was performed under a licence for experimenting on animals by the German Federal State Veterinary Administration Office in Lower Saxony (addition to study No. 33.9-42502-05-11A104, LAVES, Oldenburg, Germany).

### Selection of herds

Overall, 125 breeding herds were selected from databases of four different marketing companies trading growing and slaughter weight pigs for farmers in the North-western part of Germany. Each database comprised approximately 50 breeding herds, but not all of them met the inclusion and exclusion criteria of this study. The principle inclusion factor was the production type ‘breeding herd’. It was out of interest, whether these herds were operating as one-site production (‘farrow-to-finish herds’) or two-site production systems (selling the growing pigs). Both production types have in common that the nursery unit(s) are located at the same place where the sows and their offspring were kept. Any purchase of pigs others than gilts or a replacement boar was treated as an exclusion criterion. All 125 herds of this study met the inclusion criteria.

The herds were typically managed according to a batch-wise farrowing system in conjunction with age-segregated rearing of the weaning and growing/finishing pigs, and an all-in/all-out hygiene policy at the level of the farrowing units, nurseries and fattening pig units.

### Data collection

The information on herd level was captured with a structured and standardised questionnaire, consisting of 75 closed (e.g. yes/no) and 68 semi-closed (e.g. frequencies of procedures) questions on numerous aspects of herd management, husbandry and environment. All criteria collected were considered as potential risk factors for the occurrence of the *M. hyopneumoniae* infection in suckling pigs at the age of weaning. The questionnaire was pre-tested on ten herds by the field investigator (HW). These ten herds were not included in the intrinsic study.

All 125 herds were visited once and all questionnaires were filled out by the same investigator to avoid any interviewer variation. The investigator started the herd visit with a face-to-face interview with the producer. This was followed by an inspection of the entire pig herd where the given answers were assessed, facilitating on-site verification. Farmers´ answers not in accordance with the actual conditions were corrected by the investigator.

### Sampling

In each herd, nasal swabs were collected from 20 suckling pigs aged 18 to 24 days of life. Two piglets per litter, the lightest male and female individuals each, were selected and swabbed in the ventral nasal passages of both sides. Lightest piglets were selected in order to standardise the sampling procedure without extensive randomization procedures for each litter.

The sample size needed to detect *M. hyopneumoniae* in at least one sample per herd was calculated based on an estimated prevalence of 15% and approximately 110 piglets in the subpopulations. This estimation was based on previous findings in other studies examining the prevalence of *M. hyopneumoniae* in suckling pigs [6,11,12]. In each herd, these subpopulations were comprised by 10 litters of an appropriate farrowing batch, where piglets had been at least 18 days old. An average litter size was estimated being approximately 11 piglets per litter at the time of weaning. The level of confidence was set at 95%.

### Laboratory analysis

Subsequently to sampling, the nasal swabs were directly submitted to DNA isolation. The top of each swab was clipped and transferred into a reaction tube containing 1.5 mL Tris-EDTA (TE) buffer. After 30 min of incubation at 56ºC, the top of the swab was transferred into a shortened filter tip, which was placed in a new reaction tube, whereas the liquid in the primary tube was stored. The construct in the secondary tube was centrifuged at 18,000 g lasting 15 sec. Subsequently, the filter tip containing the top of the swab was discarded and the liquids of both reaction tubes were merged and mixed in a new one. After centrifugation at 18,000 g lasting 20 min, pellets were submitted to DNA isolation using a silica-membrane-based spin kit according to the manufacturer’s instructions (QIAamp DNA Mini kit, Qiagen). A swab previously dipped into TE buffer containing a modified *Escherichia coli* (K12 p_MYO_REP & p_MYO_ABC) served as an external positive control for successful DNA isolation and amplification in every test setup. The genetically modified strain of *E. coli* is carrying plasmids, which had been extended by the PCR target sequences of the REP and ABC genes [13]. Swabs dipped in pure TE buffer were used as negative control. The DNA was examined using real-time PCR amplifying sequences of the REP- and ABC-genes of *M. hyopneumoniae*[13]. The assay had been modified to a multiplex PCR [14] and was carried out on a AB 7500 cycler (Life-technologies).

### Statistical analysis

Results from clinical examination and laboratory testing were entered into a spread-sheet program (Excel for Office 2010, http://www.microsoft.com) and further analysed using SAS v9.1.2 (SAS Institute Inc. Cary, NC) and NCSS 2007 (http://www.ncss.com).

A herd was defined as positive for *M. hyopneumoniae* when at least one nasal swab taken from the herd had a positive PCR outcome. All potential risk factors were screened for their individual association with the binary outcome (herd *M. hyopneumoniae* positive or negative) using a logistic regression (LR) model, and results recorded as odds ratios (OR) with related 95% confidence intervals and Wald *P*-values. For categorical risk factors, class frequencies and class-specific OR were assessed, and adjacent low frequency classes merged in order to reduce the number of classes and to increase the frequencies. Interval-measured risk factors were included into the LR model (a) as continuous (numerical) information (generating one OR estimate) and (b) as categorical variables after creating frequencies by quartile ranges. If the OR estimates over the quartiles were showing a linear (increasing/decreasing) trend, the assumption of linearity was considered to be met, and the interval format was retained. Otherwise the variable entered the LR model as a categorical variable. Spearman Rank correlation coefficients between all risk factors were derived. In case of substantial correlation (r >0.50) a decision was taken which of the variables in the pair was to retain for a final model.

Five multivariable models, each including only those parameters belonging to the same ‘biological context group’ (‘Herd management’, 'Farrowing’, ‘Vaccination of piglets’, ‘Replacement policy’, ‘Vaccination of replacement pigs’) that had univariable *P*-values <0.2, were run. Adjusted OR (corrected for the influence of the other variables in the respective models) with 95% CI and *P*-values were derived. *P*-values <0.05 were considered as significant.

## Results

In this cross-sectional study 69.6% (87/125) of the herds were one-site production systems operating from farrow to finish. In contrast, 27.2% (34/125) of the herds were breeding herds, where all piglets had been sold at the end of the nursery phase. Another 3.2% (4/125) of the herds were basically piglet producers, but also reared a few pigs to market weight. The average herd size was 220 sows in the stock (median; range: 100-1,000) including 31 gilts (median; range: 1-161).

**Figure 1:**
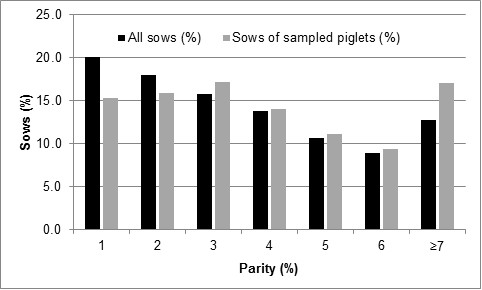
Age structure of sows within the herds and those whose piglets were sampled.

Nasal swabs for *M. hyopneumoniae* detection were obtained from piglets aged 21.8 days on average (mean; SD: 3.4). The average duration of the suckling period in these herds was 24.2 days (mean; SD: 3.0), the mean number of life born piglets in the corresponding litters was 12.7 (SD: 3.1) and the median parity of the sows of these litters was 4 with a range from 1 to ≥7 (Figure 1).

### Prevalence of M. hyopneumoniae infections in suckling pigs on herd level

In total, 3.9% (98/2500) of all nasal swabs from suckling pigs were tested positive for *M. hyopneumoniae* by real-time PCR. Piglets tested positive for specific genome fragments originated from 46 different herds resulting in an overall herd prevalence of 36.8% (46/125) for *M. hyopneumoniae* infection in suckling pigs at the age of weaning. The number of positive samples within these herds was ranging from one sample in 56.5% (26/46), two samples in 28.3% (13/46) and more than two samples in 15.2% (7/46) of the herds with a maximum of 15 positive tested samples in one herd.

### Risk factor analysis

In a first step, frequencies were analyzed (Tables 1, 2, 3). Overall, 16 parameters showed a Wald *P*-value <0.2 in the univariable approach and were therefore considered as candidates for the final multivariable analysis (Table 4). Noteworthy, 13 of these parameters demonstrated statistical significance in the univariable analysis (Wald *P*-value <0.05).

**Table 1:** Variables and their level describing general factors, management and husbandry systems

**Variable**	**Level**	**Herds per level (%)**	**Herds per level (n)**
**General herd factors**			
Number of sows	< 250	58.4	73
	250 - < 500	32.0	40
	≥ 500	9.6	12
Number of nursery pigs	≥ 250 - < 500	9.6	12
	≥ 500 - <1,000	46.4	58
	≥ 1,000	44.0	55
	0	27.2	34
Number of fattening pigs	> 1 - < 500	6.4	8
	≥ 500 - < 1,000	25.6	32
	≥ 1,000 - < 2,000	25.6	32
	≥ 2,000	15.2	19
Production or age groups within the same building as the farrowing unit	No others	15.2	19
	Nursery pigs and fattening pigs	12.0	15
	Sows	48.0	60
	Nursery-/ fattening pigs and sows	24.8	31
Moving of sows through compartments stocked with nursery pigs	No	98.4	123
	Yes	1.6	2
Moving of sows through compartments stocked with fattening pigs	No	99.2	124
	Yes	0.8	1
**Management in the farrowing unit**			
Farrowing rhythm	continuous farrowing	4.8	6
Batch-wise farrowings:	every week	11.2	14
	every second week	31.2	39
	every third week	41.6	52
	every fourth week	9.6	12
	at longer intervals than four weeks	1.6	2
All-in/all-out policy	100% of all batches	68.0	85
	< 100% of all batches	32.0	40
Farrowing unit for sows not fitting into a dedicated batch of the rhythm (RTE)	No	56.8	71
	Yes	43.2	54
Idle time of the farrowing unit in days	< 1	19.2	24
	> 1 - < 3	46.4	58
	> 3	34.4	43
Disinfection of the compartment before restocking	100% of all batches	23.2	29
	< 100% of all batches	76.8	96
Cleaning of the compartment with high pressure cleaner before restocking	100% of all batches	0.8	1
	< 100% of all batches	99.2	124
Washing of the sows before moving into the farrowing unit	never	45.6	57
	100% of all batches	32.8	41
	< 100% of all batches	21.6	27
Age of piglet in days p.p., when receiving first iron injection	< 3	79.2	99
	> 3	20.8	26
Repetition of iron injection in piglets	No	60.8	76
	Yes	39.2	49
Age of piglets in days, when castration takes place	< 3	20.8	26
	> 3 - < 7	49.6	62
	> 7	29.6	37
Age of piglets in days, when tail docking takes place	< 3	75.2	94
	> 3 - < 7	24.8	31
Age of piglets in days, when teeth grinding takes place	No teeth grinding	35.2	44
	> 1 - < 7	64.8	81
Age of piglets in days, when ear tagging takes place	< 3	12.0	15
	> 3 - < 7	40.8	51
	> 7	47.2	59
Age of piglets in days, when weaning takes place	< 20	8.0	10
	> 20 - < 28 days	87.2	109
	> 28	4.8	6
**Management in the nursery unit**			
All-in/all-out policy	100% of all batches	83.2	104
	< 100% of all batches	16.8	21
Cleaning of the compartment with high pressure cleaner before restocking	100% of all batches	100.0	125
	< 100% of all batches		
Disinfection of the compartment before restocking	100% of all batches	83.2	104
	< 100% of all batches	16.8	21
**Management in the fattening unit**			
All-in/all-out policy	100% of all batches	85.7	78
	< 100% of all batches	14.3	13
Cleaning of the compartment with high pressure cleaner before restocking	100% of all batches	90.1	82
	< 100% of all batches	9.9	9
Disinfection of the compartment before restocking	100% of all batches	69.2	63
	< 100% of all batches	30.8	28
**Replacement policy (sows)**			
Source of the gilts	Own breeding	11.2	14
	Purchase	88.8	111
Quarantine for gilts	No	5.3	6
	Yes	94.7	108
Duration of the quarantine in weeks	≤ 3	24.1	26
	> 3 - ≤ 6	66.6	72
	> 6	9.3	10
Location of the quarantine compartment	Separate farm	8.3	9
	Separate building	69.5	75
	Separate compartment	22.2	24
All-in/all-out policy	Yes	86.1	93
	No	13.9	15
Exposure to the gilts	Nothing	55.5	60
	Living pigs	30.6	33
	Faeces	13.9	15
**Replacement policy (boars)**			
Source of replacement boars	Own breeding	52.0	65
	Purchase	48.0	60
Number of purchases of replacement boars in the last 12 month	0	74.4	93
	≥ 1	25.6	32
Quarantine for replacement boars	No	78.3	47
	Yes	21.7	13
**Housing in the farrowing unit**			
Flooring in the farrowing pen	Completely plastic	26.4	33
	Other materials	73.6	92
Flooring in the piglet creep	Completely plastic	28.0	35
	Other materials	72.0	90
Heating in the piglet creep	Underfloor heating + infrared light	63.2	79
	Other systems	36.8	46
Air inlet	Door	44.8	56
	Other systems	55.2	69
Air outlet	Fan	83.2	104
	Other systems	16.8	21
**Housing of gilts**			
Flooring	Fully slatted	39.8	43
	Other systems	60.2	65
Pen walls	Concrete	57.4	62
	Open	42.6	46
Feeding system	Automatic	60.2	65
	Manual	39.8	43
Air inlet	Door	23.1	25
	Other systems*	76.9	83
Air outlet	Fan	46.3	50
	Other systems**	53.7	58
Storage of manure	Subsurface	92.6	100
	Outside of the compartment	7.4	8
**Housing of replacement boars**			
Flooring	Fully slatted	46.2	6
	Other systems	53.8	7
Pen walls	Concrete	30.8	4
	Open	69.2	9
Feeding system	Automatic	69.2	9
	Manual	30.8	4
Air inlet	Door	30.8	4
	Other systems	69.2	9
Air outlet	Fan	53.8	7
	Other systems	46.2	6
Storage of manure	Underfloor	92.3	12
	Outside of the compartment	7.7	1

*Alternative systems for air inlet include perforated ceilings, air flaps along the outer wall, air flaps in the central aisle, etc.

**Alternative systems for air outlet include centralized subsurface exhaustion, decentralized subsurface exhaustion, etc.

**Table 2:** Variables and their level describing vaccination issues

**Variable**	**Level**	**Herds per level (%)**	**Herds per level (n)**
**Vaccination against *M. hyopneumoniae (enzootic pneumonia)***			
Type of vaccine against *M. hyopneumoniae *used in **suckling pigs**	No vaccination	24.0	30
	One-shot vaccine	45.6	62
	Two-shot vaccine	30.4	33
Age of suckling pigs, when vaccinated against *M. hyopneumoniae *with a one-shot	Younger than 3 weeks	37.9	36
	3 weeks or older	27.4	26
	Two-shot vaccine in use*	34.7	33
Type of vaccine against *M. hyopneumoniae *used in **gilts**	No vaccination	70.4	88
	One-shot vaccine	20.8	26
	Two-shot vaccine	8.8	11
Frequency of vaccination of gilts against *M. hyopneumoniae*	Once	61.8	23
	Twice	38.2	14
Type of vaccine against *M. hyopneumoniae *used in **replacement boars**	No vaccination	85.6	107
	One-shot vaccine	8.8	11
	Two-shot vaccine	5.6	7
Vaccinations against PRRSV (PRRS)**			
Vaccination of **suckling pigs **against PRRSV	No vaccination	75.2	94
	Modified live virus vaccin	24.8	31
Age of suckling pigs, when vaccinated against PRRSV	Younger than 3 weeks	54.8	17
	3 weeks or older	45.2	14
Type of vaccine against PRRSV used in **sows**	No vaccination	16.0	20
	Modified live virus vaccine	84.0	105
Timing of vaccination of sows against PRRSV	Gestation oriented	25.2	27
	Mass vaccination of whole sow herd	74.8	80
Timing of vaccination of sows against PRRSV, if gestation oriented	6/60 or 5/50	44.4	12
	Other rhythm	55.6	15
Days between last vaccination of sows against PRRSV and day of herd examination	< 30	25.0	20
	> 30 - < 90	52.5	42
	> 90	22.5	18
Type of vaccine against PRRSV used in **gilts**	No vaccination	11.2	14
	Modified live virus vaccine	88.8	111
Frequency of vaccination of gilts against PRRSV	Once	31.9	36
	Twice	68.1	77
Vaccination of **replacement boars **against PRRSV	No	48.0	60
	Yes	51.2	65
**Vaccinations against PCV2 (PCVAD)**			
Vaccination of **suckling pigs **against PCV2	No vaccination	35.2	44
	Vaccination	64.8	81
Age of suckling pigs, when vaccinated against PCV2	Younger than 3 weeks	20.0	17
	3 weeks or older	80.0	68
Vaccination of **sows **against PCV2	No	85.6	107
	Yes	14.4	18
Vaccination of **gilts **against PCV2	No	63.2	79
	Yes	36.8	46
Vaccination of **replacement boars **against PCV2	No	82.4	103
	Yes	17.6	22
**Vaccinations against SIV (Influenza)**			
Vaccination of **sows **against SIV	No	48.0	60
	Yes	52.0	65
Vaccination of **gilts **against SIV	No	38.4	48
	Yes	61.6	77
Vaccination of **replacement boars **against SIV	No	66.4	83
	Yes	33.6	42
**Vaccinations against *P. multocida (progressive atrophic rhinitis)***			
Vaccination of **sows **against *Pasteurella multocida*	No	96.8	121
	Yes	3.2	4
Vaccination of **gilts **against *Pasteurella multocida*	No	96.8	121
	Yes	3.2	4
Vaccination of **replacement boars **against *Pasteurella multocida*	No	96.0	120
	Yes	4.0	5
**Vaccinations against *A. pleuropneumoniae(APP)***			
Vaccination of **sows **against APP	No	95.2	119
	Yes	4.8	6
Vaccination of **gilts **against APP	No	86.4	108
	Yes	13.6	17
Vaccination of **replacement boars **against APP	No	88.8	111
	Yes	11.2	14

* Two shot vaccines were administered at 1 and 3 weeks of age.

**No inactivated vaccines have been used.

**Table 3:** Variables and their level describing antimicrobial treatments

**Variable**	**Level**	**Herds per level (%)**	**Herds per level (n)**
Routine antibiotic treatment in suckling pigs	No	14.4	18
	Yes	85.6	107
Number of treatments in the first week of life	0	18.4	23
	1	63.2	79
	2	18.4	23
Type of drug in the first week	No treatment	18.4	23
	Effective against M. hyopneumoniae*	18.4	23
	Not effective against *M. hyopneumoniae*	63.2	79
Number of treatments in the second week of life	0	86.4	108
	1	13.6	17
Type of drug in the second week	No treatment	86.4	108
	Effective against M. hyopneumoniae*	5.6	7
	Not effective against *M. hyopneumoniae*	8.0	10
Routine antibiotic treatment in gilts	No	83.2	104
	Yes	16.8	21
Routine antibiotic treatment in replacement boars	No	98.4	123
	Yes	1.6	2
Routine antibiotic treatment in sows *ante-partum*	No	97.6	122
	Yes	2.4	3
Routine antibiotic treatment in sows *post-partum*	No	93.6	117
	Yes	6.4	8

* Treatments with Amoxicillin, Ampicillin, Colistin and Penicillin were considered being ‘not effective against *M. hyopneumoniae’*. In contrast to this, all remaining antimicrobials that were used for the treatment of animals enrolled in this study were taken into account as being ‘effective against *M. hyopneumoniae*’. These antimicrobials namely were Apramycin, Enrofloxacin, Tetracyclin and Tulathromycin.

**Table 4:** Adjusted odds ratios (OR) and 95% confidence intervals from five risk factor groups

**Model & variables**	**Level**	**Odds ratio**	**95% CI**	**p-value**
**Herd management**				
Exclusive piglet producer without any fattening pigs	Yes	0.90	0.39-2.10	0.81
	No	1.00		
Between 250 and 500 sows per herd	Yes	1.88	0.81-4.33	0.14
	No	1.00		
More than 500 sows per herd	Yes	1.61	0.40-6.59	0.50
	No	1.00		
**No batch farrowing or farrowing rhythm not in accordance with the sows’ natural oestrus cycle**	**Yes**	**2.67**	1.24-5.73	**0.01**
	No	1.00		
**Farrowing**				
Maximum number of farrowing pens per compartment: 8-16	Yes	1.81	0.74-4.42	0.19
	No	1.00		
**Maximum number of farrowing pens per compartment: > 16**	**Yes**	**3.31**	1.69-9.37	**0.02**
	No	1.00		
Air inlet in the farrowing unit not solely comprised by door inlet	Yes	1.57	0.68-3.63	0.59
	No	1.00		
Air outlet in the farrowing unit not solely comprised by door outlet	Yes	1.79	0.65-4.94	0.26
	No	1.00		
Age of piglets when ear tagging takes place: > 3 and < 7 days	Yes	2.50	0.56-11.27	0.23
	No	1.00		
Age of piglets when ear tagging takes place: > 7 days	Yes	3.74	0.68-16.23	0.08
	No	1.00		
**Vaccination of suckling pigs**				
**one-shot-vaccine against *M. hyopneumoniae***	**Yes**	**5.50**	1.62-18.65	**0.01**
	No	1.00		
**two-shot-vaccine against *M. hyopneumoniae***	**Yes**	**4.69**	1.31-16.80	**0.02**
	No	1.00		
**Vaccine against PRRSV**	**Yes**	**4.39**	1.66-11.61	**<0.01**
	No	1.00		
**Replacement policy**				
4 - 7 purchases of gilts per year	Yes	1.40	0.49-3.99	0.53
	No	1.00		
> 8 purchases of gilts per year	Yes	4.28	0.75-24.45	0.10
	No	1.00		
Total number of gilts purchased per year: 80 -119	Yes	1.09	0.38-3.14	0.88
	No	1.00		
**Total number of gilts purchased per year: > 120**	**Yes**	**5.80**	1.68-20.03	**0.01**
	No	1.00		
Age of gilts, when delivered < 180 days	Yes	1.00	0.99	1.01
	No	1.00		
**Vaccination of replacements**				
**Vaccination of gilts against PCV2**	**Yes**	**3.55**	1.31-9.63	**0.01**
	No	1.00		
Vaccination of replacement boars against *M. hyopneumoniae*	Yes	1.71	0.48-6.13	0.41
	No	1.00		
Vaccination of replacement boars against PCV2	Yes	2.38	0.62-9.06	0.20
	No	1.00		

Results were obtained from independent multivariable logistic regression models.

A reduced chance of finding *M. hyopneumoniae* in suckling pigs was recorded in all in-all out farrowing systems when employing batch-wise farrowing every week or every three weeks (OR: 0.36; *P* = 0.008). The reduction was even stronger (OR: 0.31; *P* = 0.015), when herds applying batch-wise farrowing every three weeks were compared only with herds employing a batch-wise farrowing every two weeks.

An increase in time before restocking the farrowing units with a new batch of sows prior to farrowing was a preventive measure (OR: 0.30; *P* = 0.029).

In herds, where so-called ‘one-shot’ or ‘two-shot’ vaccines against *M. hyopneumoniae* were applied to the suckling pigs, infections with the correspondent agent were found more frequently (OR: 3.64; *P* = 0.021 and OR: 3.49; *P* = 0.035, respectively). Similar results with an increased risk for suckling pigs to be infected with *M. hyopneumoniae* were seen when they received vaccines against PRRSV (OR: 2.72; *P* = 0.018). Further factors increasing the probability of detecting *M. hyopneumoniae* in nasal swabs from piglets were attributed to an air inlet into the farrowing units via the door (OR: 2.23; *P* = 0.038) and a maximum number of farrowing pens in a single compartment greater than 16 (OR: 1.06; *P* = 0.016).

Herds where either gilts or replacement boars were vaccinated against PCV2 during their acclimatization period were more likely to be classified ‘positive for *M. hyopneumoniae* in suckling pigs’ than herds not vaccinating their replacements (OR: 4.49; *P* <0.001 and OR: 6.49; *P* <0.001, respectively). Moreover, the detection of the agent in suckling pigs was associated with a high number of purchases of gilts per year (OR: 1.20; *P* = 0.015), a high ‘minimum number of gilts per purchase’ (OR: 1.03; *P* = 0.039), a high ‘maximum number of gilts per purchase’ (OR: 1.03; *P* = 0.045) and a high ‘total number of purchased gilts per year’ (OR: 1.01; *P* = 0.005).

From the five multivariable models, seven variables (‘Batch-wise farrowing system used in the herd’, ‘Number of farrowing pens per compartment’, ‘Vaccination of gilts against PCV2’, ‘Number of gilts purchased per year’, ‘Use of one-shot-vaccines against *M. hyopneumoniae* in suckling pigs’, ‘Use of two-shot-vaccine against *M. hyopneumoniae* in suckling pigs’ and ‘Vaccination of sucking pigs against PRRSV’) could be identified being significantly associated with the detection of *M. hyopneumoniae* in suckling pigs at the time of weaning (Table 4).

## Discussion

A cross-sectional study including 125 herds was performed evaluating the prevalence of *M. hyopneumoniae* infections in suckling pigs at the age of weaning and the potential of corresponding risk factors on herd level.

The region where this study was conducted has the highest pig density in Germany (> 500 pigs/ 100 hectare agricultural used space; [15]), characterized by typical configurations of intensive pig production. Herd structures, husbandry systems and management procedures assessed during this investigation are probably highly representative for many other regions in Europe with intensive pig production. In order to minimize potential selection bias, the herds enrolled in this study were not proposed by field veterinarians but were randomly selected from databases of marketing companies. The participation in the study was voluntary, but all producers asked were willing to participate. The unexpectedly high motivation may be the result of a feeling of obligation to and a degree of economic dependence on the marketing company. Furthermore, producers were highly interested in the diagnostics and veterinary consultancy free of charge.

The transmission of *M. hyopneumoniae* cannot securely be prevented by vaccinating piglets or pigs against this pathogen [16]. This lack of highly effective vaccines demands for additional actions reducing the impact of *M. hyopneumoniae* infections on the health of pigs. Any measures lowering the proportion of suckling pigs infected by their mother sow are supposed to decrease the later spread of the agent among growers and finishers [9,17]. Whether these vertical transmissions of *M. hyopneumoniae* and corresponding infections in suckling pigs occur with high frequencies and, therefore, have to be considered in prevention programmes is intensively discussed. Studies conducted in single endemically infected herds or herds with a recent epidemic infection with *M. hyopneumoniae* revealed low prevalences of 1.5 to 3.8% in suckling pigs and 4.4 to 7.2% in nursery pigs [11], 2.6 to 13.2% in piglets 3 weeks of age [10] and 9.6% in suckling pigs prior to weaning [6]. In a retrospective study including data from more than 300 herds, comparable low prevalences of 2.0% in suckling pigs and 9.3% in nursery pigs were found [12]. In contrast to these studies, rates of over 30% have been reported previously in suckling pigs, but these studies included only a limited [17,18] or an unknown number of herds [19].

In the present study, 3.9% (98/2,500) of all suckling pigs and 36.8% (46/125) of all herds were tested positive for *M. hyopneumoniae*. A real-time PCR has been used to detect the pathogen in nasal swabs from suckling pigs facilitating a high sensitivity. This PCR assay showed 85 to 90% sensitivity on pig level, when lung tissue was examined and both target sequences (ABC and REP) were amplified [13]. Notwithstanding, it has also been used to determine *M. hyopneumoniae* infections by testing nasal swabs [20]. Taking this into account, there is evidence that both figures, the overall detection rate among suckling pigs and the prevalence of herds showing *M. hyopneumoniae* infections in this age group, are good estimates for the current situation in the pig population of Northern Germany. Indeed a true prevalence of 3.9% would require 58 samples per herd in order to detect *M. hyopneumoniae* in at least one piglet out of a group of 110 piglets, but of course this sampling fraction is not affordable and the reasonable sample size used in the present study was already bigger than in other studies. Thus, the sample size was a good compromise, even though the sampling scheme as applied is missing a maximum possible prevalence of 12.7%, when all collected samples are tested negative.

Noteworthy, the probability of detecting *M. hyopneumoniae* in suckling pigs was lower than reported earlier [12], but it seems to be possible in approximately one third of all herds, when the sample size is large enough. These low detection rates are not in contrast to the hypothesis that single infected suckling pigs may act as spreader in the subsequent nursery or growing units [10].

In the multivariable analysis several herd factors have been identified being associated with the detection of *M. hyopneumoniae* in suckling pigs at the time of weaning. Whereas three factors could be assigned to risk factors, four others are more likely to be a result than a reason of frequent infections in suckling pigs: herds had a higher chance of at least one positive piglet, when ‘one-shot’ vaccines (OR: 5.5) or ‘two-shot’ vaccines (OR: 4.7) against *M. hyopneumoniae* were applied to the suckling pigs. Moreover, the risk was significantly increased, when piglets were vaccinated against PRRSV (OR: 4.4) or gilts were vaccinated against PCV2 (OR: 3.6). It is hypothesised that enzootic pneumonia in fattening pigs is eventually complicated by PRRS, both reflecting endemic infections in the herd including a higher probability of vertical transmission of the pathogens from sows to their offspring, drives the farmer to the decision of vaccinating suckling pigs against *M. hyopneumoniae* and/or PRRSV. Similar interactions can be assumed for PCV2 and gilt vaccination. The association between *M. hyopneumoniae* infections and the application of different vaccines was even expected, since their use is often an economic incitement for owners of endemically infected farms.

The risk of a herd to have a *M. hyopneumoniae* infection among suckling pigs was increased when the total number of purchased gilts per year was higher than 120 (OR: 5.8). This observation underlines the necessity of an appropriate acclimatisation period for replacements pigs, which should last approximately four to six weeks [5]. At a certain time point, gilts should get in a close nose-to-nose contact to pigs from the stock herd [21], because transmission of *M. hyopneumoniae* by other vectors is insufficient for the purpose of provoked exposure [22]. It should be mentioned that sentinels for nose-to-nose contact must not re-enter the stock herd, when they developed any symptoms of infectious disease during the contact phase. Therefore, the use of old sows or nursery pigs with stunted growth, dedicated for slaughter, are highly recommended as sentinels.

The number of sows housed in compartments of the farrowing units was also associated with the outcome variable. The risk of a herd being positive in suckling pigs significantly increased, when the number of farrowing pens in one compartment was higher than 16 (OR: 3.3). Again there is doubt, whether all-in/all-out policy is strictly implemented, when farrowing units are operated with a high number of pens per compartment, although this factor is known having major impact on the prevention of transmission of *M. hyopneumoniae* within herds [23]. Beside this, a higher number of pens per compartment also increase the probability of housing gilts in their first parity together with older sows. These gilts shed *M. hyopneumoniae* more often than older sows [3,6] and by doing this enable a frequent transmission of the pathogen to their own offspring, but also to piglets of other litters via aerosol [24].

Herds, where no batch farrowing or any kind of batch-wise farrowing rhythm that does not allow simple integration of sows returning to oestrus (i.e. not 1- or 3-week rhythm) was implemented, were more often found to be positive (OR 2.7). This observation was in accordance with another study reporting higher seroprevalences towards *M. hyopneumoniae* in sows farrowing in a 2- or 4-week rhythm [7]. When comparing only herds with 2-week farrowing-rhythm to those with 3-week rhythm, the effect was even stronger (OR: 3.8). It can be assumed that in such herds, especially those with 2-week rhythm, a strict realisation of the all-in/all-out policy for the farrowing units is not possible and not performed, because sows which have regularly returned to oestrus approximately 21 days after insemination neither fit into the last nor into the next group. Conclusively, such sows will farrow one week earlier or one week later compared to all other sows, and if the herd does not have appropriate compartments for sows farrowing ‘out of the rhythm’ these sows will be allocated into compartments, where other sows have already farrowed or will farrow later. Taking this fairly conclusive assumption into account, it seems to be the non-compliance with the all-in/all-out policy rather than the farrowing interval that increases the likelihood of *M. hyopneumoniae* positivity in suckling pigs at the age of weaning.

## Conclusions

In this cross-sectional study, several risk factors (more than 120 replacement gilts purchased per year, farrowing compartments with more than 16 pens, no batch farrowing or batch farrowing rhythm not easy to synchronize with the sows’ natural oestrus cycle, i.e. 2- or 4-week rhythm) could be identified enhancing the probability of breeding herds to raise suckling pigs already infected with *M. hyopneumoniae* at the time of weaning. Interestingly, some factors (farrowing rhythm, gilt acclimatisation issues) were overlapping with those also influencing the seroprevalences among sows or the transmission of the pathogen between older age groups. Taking the multifactorial character of enzootic pneumonia into account, the results of this study substantiate that a comprehensive herd specific prevention programme is a prerequisite to reduce transmission of and disease caused by *M. hyopneumoniae*.

## Competing interests

The authors have no financial or personal relationship with people or organisations that could inappropriately influence or bias the content of this paper.

## Authors’ contributions

EGB, MGD and HN designed the cross-sectional study and HW and SD carried out the farm visits and data collection. HW and SD performed the laboratory tests under supervision of HN. MGD assisted with statistical analysis and ASF performed statistical analysis. HN drafted the manuscript. ASF, MGD and EGB critically revised the manuscript. All authors read and approved the final manuscript.
